# Molecular Remodeling of Cardiac Sinus Node Associated with Acute Chagas Disease Myocarditis

**DOI:** 10.3390/microorganisms9112208

**Published:** 2021-10-23

**Authors:** Héctor O. Rodríguez-Angulo, Diana Colombet-Naranjo, María C. Maza, Cristina Poveda, Alfonso Herreros-Cabello, Iván Mendoza, Juan C. Perera, Juan D. Goyo, Núria Gironès, Manuel Fresno

**Affiliations:** 1 Instituto Venezolano de Investigaciones Científicas, Caracas 1020A, Venezuela; hector.rodriguez@ucla.edu.ve (H.O.R.A.); colombet.diana@gmail.com (D.C.-N.); 2 Unidad de Biología Celular, Departamento de Ciencias Morfológicas, Programa de Medicina, Facultad de Ciencias de la vida, Universidad Centroccidental Lisandro Alvarado, Barquisimeto 3001, Venezuela; juancaperecas@gmail.com (J.C.P.); escalonagoyojuandediego@gmail.com (J.D.G.); 3 Departamento de Biología Molecular, Universidad Autónoma de Madrid, 28049 Madrid, Spain; mcma-za@cbm.csic.es (M.C.M.); cristinapovedac@gmail.com (C.P.); alfonso.herreros@uam.es (A.-H.C.); 4 Centro de Biología Molecular Severo Ochoa, CSIC-UAM, 28049 Madrid, Spain; 5 Instituto de Medicina Tropical, Universidad Central de Venezuela, Caracas 1060, Venezuela; imivanjm@gmail.com; 6 Instituto de Investigación Sanitaria Hospital Universitario de la Princesa, 28009 Madrid, Spain

**Keywords:** *Trypanosoma cruzi*, chagas disease, sudden death, electrocardiograms, heart rate, arrhythmias, HCN4, ivabradine, isoproterenol, β2-adrenergic receptor

## Abstract

Chagas disease principally affects Latin-American people, but it currently has worldwide distribution due to migration. Death among those with Chagas disease can occur suddenly and without warning, even in those who may not have evidence of clinical or structural cardiac disease and who are younger than 60 years old. HCN4 channels, one of the principal elements responsible for pacemaker currents, are associated with cardiac fetal reprogramming and supraventricular and ventricular arrhythmias, but their role in chagasic arrhythmias is not clear. We found that a single-dose administration of ivabradine, which blocks HCN4, caused QTc and QRS enlargement and an increase in P-wave amplitude and was associated with ventricular and supraventricular arrhythmias in mice challenged with isoproterenol, a chronotropic/ionotropic positive agent. Continuous treatment with ivabradine did not alter the QTc interval, but P-wave morphology was deeply modified, generating supraventricular arrhythmias. In addition, we found that repolarization parameters improved with ivabradine treatment. These effects could have been caused by the high HCN4 expression observed in auricular and ventricular tissue in infected mice. Thus, we suggest, for the first time, that molecular remodeling by overexpression of HCN4 channels may be related to supraventricular arrhythmias in acute Chagas disease, causing ivabradine over-response. Thus, ivabradine treatment should be administered with caution, while HCN4 overexpression may be an indicator of heart failure and/or sudden death risk.

## 1. Introduction

Chagas disease is a tropical illness that affects 6–8 million people throughout Mexico and Central and South America and, in recent times, it has been spread worldwide due to human migration [1]. Typically, the disease course involves an acute phase, often oligosymptomatic but on some occasions with a fatal outcome, and a chronic phase that is symptomatic in only 10–30% of cases [2]. Sudden death has been reported as the principal cause of mortality, with nearly 50% of cases involving previously asymptomatic fatal episodes [3,4]. A possible cause of sudden death is myocardial disarray related to adrenergic stress [5]. One of the most challenging points of Chagas disease research is elucidating the causes of disease progression and fatality during the chronic phase, especially sudden death and heart remodeling pathophysiology. Previous reports by our team related arrhythmogenesis with parasite-secreted proteins [6], the parasite secretome being linked with virulence and cell invasion [7], reinforcing the possible role of *Trypanosoma cruzi* proteins in the pathogenesis of arrhythmias. On the other hand, autoimmunity has been linked to arrhythmias during ongoing Chagas disease. Specifically, the M2 muscarinic [8] and β2-adrenergic [9] receptors have been reported to be targets of autoantibodies during the chronic phase and possibly related to ventricular arrhythmias. Interestingly, nodal dysfunction in chagasic patients showed a positive correlation with anti-muscarinic and anti-β2-adrenergic autoantibody circulating levels [10,11], suggesting a possible link between nodal cell signaling and electrical disturbance in the pacemaker.

Ion channel dysfunction has been associated with sudden death in several familiar pathologies [12,13]. Additionally, there is evidence concerning the role of ion-channel functionality and heart disease. Hyperpolarization-activated, cyclic nucleotide-gated (HCN) ion channels, responsible for the so-called pacemaker or funny currents (I*f*) and involved in the generation of pacemaker potential in heart-sinus nodal cells, have gained importance in the understanding of sudden death and heart failure. HCN channels show four isoforms (HCN1–4), HCN4 being one of the most represented in cardiac muscle [14]. Ion channel mutations have been pointed out as causes of a hereditable syndrome associated with sudden death in patients with Brugada syndrome who showed a mutation in the S6 domain of the HCN4 channel [15] and associated with sinus node dysfunction related to G protein subunit β2 protein mutations [16]. For Chagas disease, there is little information about the possible role of ion channel disturbances on ventricular cardiac arrhythmias. Alterations in anion conductance have been reported in cardiac cells infected by *Trypanosoma cruzi* [17], but studies about the role of specific ionic channels are scarce. Along these lines, this study aimed to determine the pattern of HCN4 channels’ expression in auricles and ventricles of experimental acute infection and their role in nodal regulation, as evaluated by surface electrocardiogram.

To evaluate the activity of the HCN channels, responsible for I*f* currents both in control and chagasic mice, we used the specific and heart rate-dependent blocker ivabradine and evaluated the reduction of heart rate in both experimental groups. Additionally, isoproterenol, which indirectly activates (opens) HCN channels through β2-adrenergic signaling, was used to compare the ivabradine response between groups under positive chronotropic effect conditions, as ivabradine shows the highest affinity when the channel is in an open state. In this sense, stimulation with isoproterenol allows the maximization of channel open-state probability and is an approach that can be used to determine the differential response between healthy and infected mice.

## 2. Materials and Methods

### 2.1. Parasite Strains

The *T. cruzi* Y (TcII) strain was obtained from Dr. J. David (Harvard Medical School, Boston, MA, USA). The *T. cruzi* Dm28c (TcI) and VFRA (TcVI) strains were obtained from Dr. M. Miles (London School of Hygiene and Tropical Medicine, London, UK) through the European program ChagasEpiNet.

### 2.2. Ethics Statement

Experiments in Venezuela were performed in strict accordance with the Bioethics and Biosafety Norms (3rd edition) and approved by the Fondo Nacional de Ciencia y Tecnología de Venezuela (FONACIT), the Ministerio de Ciencia y Tecnología of Venezuela (2011), the Asociación Venezolana para la Ciencia de los Animales de Laboratorio, and the International Ethical Standards for Research Biomedical in Animals of the WHO (1982); animal handling and experimental procedures were approved by the Comité de Bioética Institucional (permit DIR-0031/1582/2017). Experiments in Spain were performed in strict accordance with the European Commission legislation for the protection of animals used for scientific purposes (directives 86/609/EEC and 2010/63/EU). Mice were maintained under specific pathogen-free conditions at the CBMSO (CSIC-UAM) animal facility. The protocol for the treatment of the animals was approved by the Comité de Ética de Investigación of the Universidad Autónoma of Madrid, Spain (permits CEI-14-283 and CEI-47-899. Animals had unlimited access to food and water and after the studies were euthanized by pentobarbital overdose (500 mg/kg) in a CO_2_ chamber with every effort made to minimize their suffering.

### 2.3. Experimental Infection

Six- to eight-week-old male BALB/c mice, housed in Plexiglas cages or ventilated racks, with water and commercial food ad libitum and 12-h dark/light cycles, were infected with 1000–2000 trypomastigotes of *T. cruzi* Y strain or 2000 trypomastigotes of the Dm28c and VFRA *T. cruzi* strains via intraperitoneal (IP) injection. Parasitemia was monitored every two days during the early acute (EA) phase (14–17 days post-infection).

### 2.4. Pharmacological Protocols

Experiments for ECG analysis with a single dosage of ivabradine (5 mg/kg) were run in control mice and mice infected with the Y strain (*n* = 4). Additionally, 12 animals were treated daily via IP injection with ivabradine (5 mg/kg) for up to 14 days to assess the effect of continuous treatment on ECG parameters and infection evolution.

### 2.5. Surface Electrocardiogram

Mice were previously anesthetized with a single bolus of 25 mg/kg of pentobarbital and 25 mg/kg of ketamine via IP injection. Electrocardiography (ECG) was performed using a bipolar system in which the electrodes were placed subcutaneously at the xiphoid cartilage (positive electrode), right shoulder (negative), and left shoulder (reference). Electrodes were connected to a Bioamp amplifier (AD Instruments, Bella Vista, Australia) and digitalized through an A/D converter PowerLab 8 sp (AD instruments). Digital recordings were analyzed with Chart software for Windows v7.3.1 (AD Instruments). Events were registered to 1 K/s and filtered to 60 Hz. Continuous ECG recordings were obtained to determine the basal heart rate, defined as the point where the values did not vary by more than 5%. At this point, 1.1 mg/kg of isoproterenol, a non-selective β2-adrenergic agonist with positive chronotropic effects, was added via IP injection to mice continuously treated with ivabradine and untreated mice, and the register was followed until the heart rate began to decrease. Heart rate variation concerning isoproterenol previous values was determined and wave morphology was recorded.

### 2.6. Immunofluorescence

Mice were sacrificed in a CO_2_ chamber at the EA phase. The heart was quickly removed, sagittal four-chamber sections were obtained and washed in commercial saline, and the portion destined for immunofluorescence was placed in a 4% formaldehyde solution overnight. Then, it was incubated with 30% sucrose overnight and preserved at −80 in a commercial cytomatrix until incubation. Frozen auricle slices were permeabilized with Triton X-100 (0.1%) and labeled with goat anti-HCN4 antibodies (Santa Cruz Biotechnology, Dallas, TX, USA) for 1 h (1:250), followed by secondary rabbit anti-goat-647 (Santa Cruz Biotechnology) for 1 h (1:500), DAPI (Sigma-Aldrich, Saint Louis, MO, USA) (1:10,000), and anti-connexin 43 (Santa Cruz Biotechnology) with secondary antibody-555 (Santa Cruz Biotechnology) (Cx43; 1:250) for nuclei/fiber counterstaining, respectively. Finally, slides were covered with Permount mounting medium (Thermo Fisher Scientific, Madrid, Spain) and analyzed in a high-velocity confocal microscope A1R+ (Nikon) coupled to an Eclipse TI-E inverted microscope (Nikon, Melville, NY, USA) with fixed conditions for the exposition time and laser gain for quantitative analysis.

### 2.7. Western Blot

A group of five control mice and four groups of five mice infected with 2000 trypomastigotes of the *T. cruzi* Y strain were sacrificed at 14 dpi. Heart samples reserved for Western blotting were immediately stored at −80 until extraction of proteins. Equal amounts of individual protein extracts for each mouse were pooled together for each group. Then, 20 µg of the pooled tissue extracts was resolved in 10% SDS-PAGE gel and transferred to 0.22 µm pore nitrocellulose membrane (BioRad, Madrid, Spain), which was incubated with a 1:1000 dilution of the anti-HCN4 antibody (Santa Cruz) and subsequently incubated with a 1:2000 dilution of horseradish peroxidase-conjugated secondary rabbit anti-goat antibody (Santa Cruz). HCN4 bands were visualized using ECL reagent (BioRad) after autoradiography exposure. The membrane was stained with Ponceau red as a protein-loading control.

### 2.8. Quantitative RT-PCR in Heart Tissue

Groups of mice were infected with 2000 trypomastigotes of the Dm28c, VFRA, and Y strains (*n* = 5 each group). Hearts were elicited and 25–35 mg of heart tissue was cut using a sterile scalpel blade and disrupted using a PT 1300D homogenizer (Kinematica Polytron, Thermo Fisher Scientific, Madrid, Spain). Purified RNA was obtained using NZYol (NZYTech) following the directions of the manufacturer quantified in a Nanodrop (Thermo Fisher Scientific, Madrid, Spain), and kept at −80 °C. Reverse transcription of total RNA was performed using the High Capacity cDNA Archive Kit (Applied Biosystems, Madrid, Spain) and gene amplification was performed using *Hcn4* (forward 5′-cacgacctcaactcaggcg-3′ and reverse 5′-cagcggggtccatataacag-3′) and *18s* (forward 5′-ccatccaatcggtagtagcg-3′ and reverse 5′-aacccgttgaaccccatt-3′) oligonucleotides in triplicate reactions with GoTaq qPCR Master Mix (Promega, Madrid, Spain) on an ABI PRISM 7900 HT instrument (Applied Biosystem, Life Sciences). The relative quantity of each gene expression was calculated using the comparative threshold cycle (C_T_) method following the protocol instructions. *Hcn4* quantifications were normalized to the ribosomal mouse *18s* gene to account for the variability in the initial concentration of RNA and the conversion efficiency of the reverse transcription (∆C_T_). Finally, all data from samples taken from mice were normalized to the values obtained from non-infected mice (∆∆C_T_). The relative quantity (RQ) or fold change was calculated as RQ = 2^−∆∆C^_t_.

### 2.9. Statistical Analysis

Data were analyzed using an unpaired Student’s t-test, with a one-way ANOVA post-test for trends (* *p* ≤ 0.05 and ** *p* ≤ 0.01). Linear regression was performed to test differences in the slope when the Δ heart rate and QT interval were related to the initial heart rate at ivabradine treatment or the RR interval during isoproterenol treatment, respectively. Differences were tested considering the probability of point coincidence assuming identical slopes, and a probability lower than 5% was chosen as the significance threshold. The analysis was performed using GraphPad Prism version 6.00 for Windows (GraphPad Software, San Diego, CA, USA, www.graphpad.com, accessed on 23 August 2021).

## 3. Results

### 3.1. Heart Rate Kinetics Analysis

**Figure 1:**
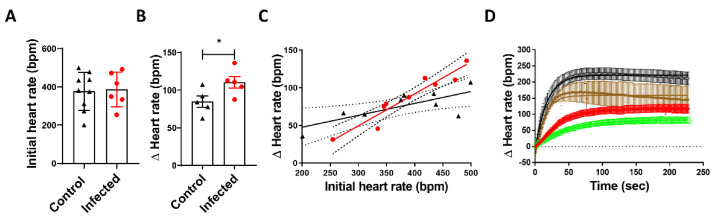
Heart rate kinetics in mice. (**A**) Initial heart rate in beats per min (bpm) in control (black symbols) and infected (red symbols) mice. (**B**) The Δ heart rate, defined as the differential of the heart rate at point 0 and 5 min post-ivabradine single-dose treatment, in control (black symbols) and infected (red symbols) groups. * The *p*-value was estimated at 0.0425 by Student’s *t*-test. (**C**) Linear regression between initial heart rate and the Δ heart rate in control (black symbols) and infected (red symbols) groups. Slopes were considered significantly different with a *p*-value of 0.0036. (**D**) Control mice were treated (brown symbols) or not treated (black symbols) with a single bolus of 5 mg/kg of ivabradine five minutes before stimulation with 1.1 mg/kg isoproterenol; infected mice were treated (green symbols) or not treated (red symbols) with ivabradine, like the control mice.

Heart rate kinetics were analyzed in control and infected mice at the early acute (EA) phase of *Trypanosoma cruzi* infection. To evaluate the sensitivity of mice to ivabradine treatment, a unique 5 mg/kg bolus treatment was administered. The infection did not significantly change the initial heart rate (Figure 1A). The Δ heart rate post-ivabradine, defined as the differential reduction of the heart rate (beats per minute (bpm)) within 5 min post-treatment with regard to the basal cardiac rate, was significantly higher in the treated group compared to the control (Figure 1B). Ivabradine is a channel blocker that shows a higher affinity for open channels [18]. Thus, as expected, a directly proportional relation was observed between the basal heart rate and Δ heart rate in both infected and healthy mice. However, we graphed the initial heart rate versus the Δ heart rate of each mouse and calculated the linear regression. A significant increase in the slope of infected mice’s linear regression was found, suggesting a higher response to ivabradine treatment in infected mice at the same heart rate (Figure 1C). Additionally, the positive chronotropic response to isoproterenol post-ivabradine treatment was evaluated up to ~230 s. Plausibly due to the stronger response to ivabradine in infected mice, the Δ heart rate after isoproterenol treatment was lower in this group in comparison to the control group with or without isoproterenol treatment (Figure 1D).

### 3.2. ECG Evaluation

**Figure 2:**
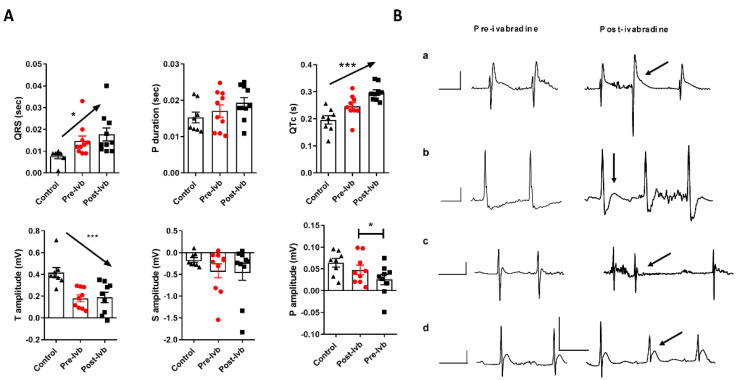
Quantitative/qualitative analysis of ECG parameters after a single-dose treatment with ivabradine (Ivb) in mice at the EA phase of infection. (**A**). ECG intervals (QRS, P duration, and QTc) and amplitudes (T, S, and P) were determined for control and infected mice before (Pre-Ivb) and after (Post-Ivb) treatment with ivabradine. An unpaired Student’s *t*-test was performed for statistical comparison (* *p* < 0.05), and a one-way ANOVA post-test for trends indicated in arrows (* *p* ≤ 0.05 and *** *p* ≤ 0.001). (**B**) Representative ECG recordings pre- and post-Ivb treatment. Registers a–d were taken from different infected mice, where a indicates a junctional ectopic beat (arrow), b the QRS enlargement with S blockage, and c–d illustrate atrial ectopic beats.

Next, the effects of single-dose ivabradine treatment on principal ECG parameters and trace morphology were analyzed. Parameters related to ventricular conduction (QRS interval), auricular conduction (P duration), ventricular repolarization (Bazzet’s heart rate-corrected QT or QTc interval, T and S amplitudes), and auricle depolarization (P amplitude) were addressed to evaluate the effects of ivabradine treatment on infected mice. Ivabradine induced QTc and QRS segment prolongation and increased P-wave voltage in the infected group, without effects on T and S voltage or P duration (Figure 2A). The morphology of ECG waves reflected these changes. Junctional ectopic beats were favored by repolarization disturbances generated by ivabradine treatment (Figure 2B, panel a) and intraventricular conduction was delayed (Figure 2B, panel b). Finally, atrial premature beats were detected post-treatment (Figure 2B, panels c and d).

**Figure 3:**
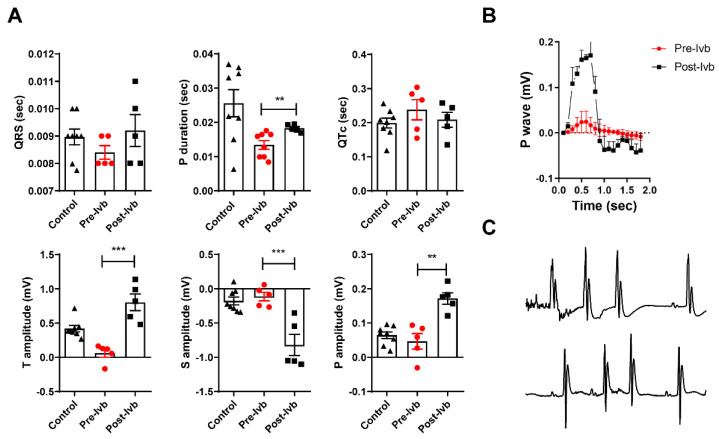
Quantitative/qualitative analysis of ECG parameters after continuous treatment with ivabradine (Ivb) in mice at the EA phase of infection. (**A**) Differences in wave voltages and amplitudes between treated (Post-Ivb) and untreated (Pre-Ivb) mice. Statistical significance was analyzed by one-way ANOVA (** *p* < 0.01 and *** *p* < 0.001). (**B**) Bimodal P-wave morphology, as seen in the mean P-wave trace. (**C**) Supraventricular ectopic beats were registered after continuous ivabradine treatment in chagasic mice.

Similar to single-dose treatment, after continuous ivabradine treatment for 17 days, a sharp increase in P-wave voltage (Figure 3A,B) accompanied by an increase in P-wave duration due to bimodal morphology was observed (Figure 3A,B). Interestingly, significant differences in S and T amplitudes without QTc increasing were observed in continuous treatment in acute contrast with single-dose treatment. Finally, supraventricular ectopic beats were detected in treated mice (Figure 3C).

### 3.3. Histological Sections

**Figure 4:**
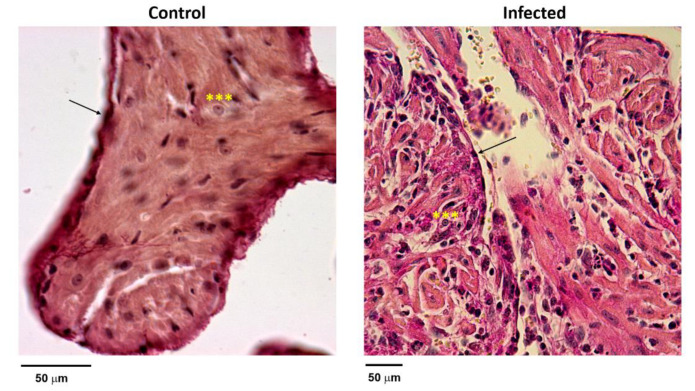
Histological sections of auricles stained with Van Gieson’s stain. The left panel shows the normal morphology of cells compatible with pyramidal P cells (***) and peripheral fusiform fibroblast-like cells (arrow) located externally to the nodal area. Structural relationships are well-defined and conserved. The right panel shows the right auricle of the infected tissue with loss of structure and depopulation of pyramidal cells. A moderate mononuclear infiltrate can be appreciated. Fusiform fibroblast-like cells preserve morphology and location (arrow).

Histological sections of right auricles of hearts from control and infected mice at the EA phase of infection, stained with Van Gieson’s stain, were evaluated for structure and cellularity. Fibroblast-like cells and pyramidal euchromatic cells compatible with nodal P cells’ reported morphology [19] were observed in healthy animal sections (Figure 4). Moderate mononuclear inflammatory infiltrates, loosening of structural architecture, and depletion of pyramidal cells were observed in infected animal auricle sections, suggesting a functional compromise of nodal function during acute Chagas disease.

### 3.4. HCN4 Expression in Heart Tissue

**Figure 5:**
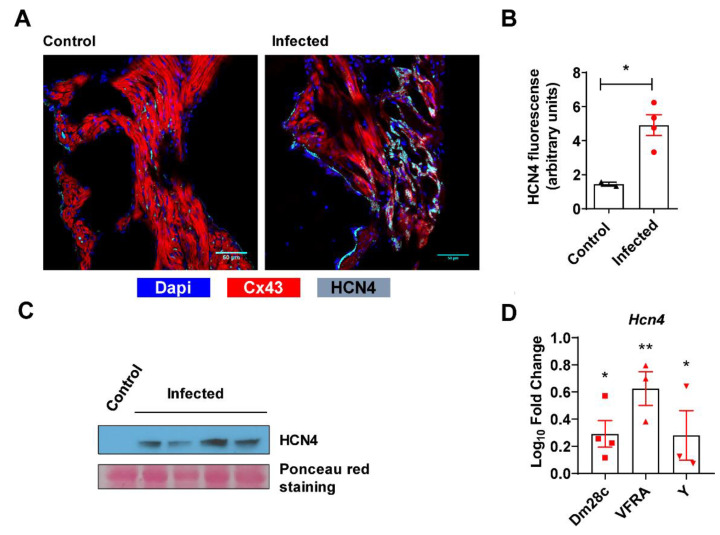
HCN4 expression in heart tissue. (**A**) Confocal immunofluorescence staining of auricles from control and infected mice at the EA phase using anti-Cx43 and anti-HCN4 antibodies, along with DAPI for nuclear visualization and to delimitate structural relationships in the nodal region of the right auricle. (**B**) HCN4 mean fluorescence was determined in control and infected samples. (**C**) Western blot analysis of ventricles was performed to estimate HCN4 expression in control and infected mice at the EA phase. The membrane was stained with Ponceau red before incubating with antibodies as a protein load control. (**D**) *Hcn4* mRNA expression in hearts of control and infected mice at the EA phase. Fold change in each infected group was calculated compared to control (“0” value in log 10 scale). Statistical analysis was undertaken using an unpaired Student’s t-test of the infected and the control groups in B and D (* *p* < 0.05; ** *p* < 0.01).

The following step was quantification of HCN4 channel expression. Auricles were evaluated by confocal immunofluorescence microscopy for HCN4 expression (Figure 5A) and fluorescence intensity was quantified, showing that HCN4 was overexpressed in infected auricles (Figure 5B). Additionally, HCN4 expression was estimated in ventricles using Western blot analysis (Figure 5C). As expected, control tissue did not show expression of this subunit but, interestingly, expression was detected in ventricles of infected mice, showing an ectopic localization of HCN4 in heart tissue. Moreover, *Hcn4* mRNA expression was analyzed by quantitative reverse transcription PCR (RTqPCR) in the hearts of control mice and mice infected with the *T. cruzi* Dm28c, VFRA, and Y strains, confirming the strain-independent increase in *Hcn4* expression at the EA phase (Figure 5D).

## 4. Discussion

Cardiac arrhythmias represent the principal cause of death in Chagas disease. Classically, humoral autoimmunity [20], pro-inflammatory profile [21], gap-junction malfunction [22], and cardiac structural changes, among other causes, have been related to sudden death in Chagas disease. For other malignant arrhythmia-causing cardiopathies, there are several reports which involve cardiac ion-channel dysfunction in the genesis of cardiac electrical disturbances [23,24]. For Chagas disease, disorganization (Cx43) [25] and mechanical remodeling [26] of gap-junction protein connexin 43 have been implied in chagasic cardiomyopathy and arrhythmias. However, the role of ion channels has not been fully addressed in Chagas disease. Following this line of thought, this work explored the possible role of HCN4 in acute chagasic myocarditis.

Histological data, in concordance with the previous report from our group [27], showed a moderate auricular inflammatory reaction with Y strain infection (Figure 5). Although no evidence of the infection is shown here, parasite load was quantified in the same cardiac samples in a previously published manuscript [28], indicating cardiac parasitization. The right atrial cell population was evaluated on this occasion with more detail, suggesting nodal-like cell depopulation. These data coincide with the fact that sick sinus syndrome, atrial extrasystoles, intra-atrial conduction disturbances, and atrial fibrillation or flutter are common findings at different stages of the disease [29]. The pathophysiology of sick sinus syndrome is not yet clear and the causes are multifactorial, including genetics, pharmacological, structural, and inflammatory causes [30]. Interestingly, inflammation has been associated with HCN channels overexpression in bladder hypermotility [31], and with rat dorsal root ganglion neurons [32], allowing us to speculate that this association may be present in the Chagas model of atrial inflammation.

We evaluated the pharmacology response to ivabradine during the early acute phase of Chagas disease. The increasing sensitivity to typically ivabradine-dependent heart rate inhibition strongly suggests variation in affinity to HCN4 channels and/or their overexpression (Figure 1A), despite the typical adrenergic desensitization observed in chagasic mice (Figure 1B). This ivabradine over-response may have been associated with the QTc enlargement and ectopic activity observed in treated mice (Figure 2). Ivabradine inhibits the human ether-a-go-go-related gene (*hERG*) that encodes the pore-forming subunit of the rapid component of the delayed rectifier K^+^ channel and *torsades de pointes* in patients [33], although other studies failed to observe increasing QT when heart rate correction was performed [34]. Intriguingly, recent studies have reported anti-arrhythmic effects of ivabradine in short QT arrhythmias induced by ouabain, a drug used to treat some arrhythmias [35], reinforcing the possible pro-arrhythmogenic long-QT potential of ivabradine in Chagas disease.

However, important differences were observed with continuous ivabradine treatment during the EA phase of infection (Figure 3A) in P voltage and duration and T-S wave voltages. Non-significant changes were detected in QTc and, in sharp correlation, ventricular arrhythmias were not detected. On the contrary, the bimodal P wave (Figure 3B) seems to facilitate supraventricular ectopic beats. These results suggest changes in response induced by continuous treatment, especially regarding long-QT inducibility, and contrast with the possibility that tissular ivabradine accumulation predisposes to delayed action potential [36]. It is possible to speculate about the role of the repolarization reserve, experimentally associated with a minority of slowly activating components (IKs) of the delayed rectifier K^+^ current [37], in the change between acute and chronic response to ivabradine treatment in chagasic mice. The rapid delayed rectifier K^+^ current (IKr) is principally conducted by ivabradine-inhibited hERG channels and is involved in action potential duration [38]. Further studies are necessary to elucidate the pathophysiological mechanisms associated with QTc enlargement due to ivabradine treatment.

Our results concur with recent work that has warned about the pro-fibrillatory effects of ivabradine in patients with stable angina [39]. This should be especially considered for Chagas disease where, to the best of our knowledge, there are no reports about possible modulation of ECG morphology by ivabradine. Moreover, it is also important to highlight the effect of treatment on ECG parameters associated with heart perfusion, such as the S-T segment. Due to the complexity of repolarization in mouse ECG, it is not easy to establish a clear relationship with ischemic events, but the probable association of the anti-angina potential of ivabradine treatment [40] with the improvement of ventricular contraction efficiency has been convincingly described. Furthermore, microvascular pathology has been described for Chagas disease [41], suggesting that ivabradine treatment could potentially improve coronary circulation.

Finally, in striking concordance with electrophysiological and pharmacological data, we observed overexpression of the HCN4 channel in auricular and especially in ventricular tissue using Western blotting (Figure 5). As far as we know, this is the first report of HCN4 overexpression in Chagas disease. It was reported for hypertrophic cardiomyopathy, with particular emphasis on the abnormal ventricular expression of HCN4, the predominant isoform of human and mouse auricles [42]. Additionally, in a mouse model of heart failure, cardiac-specific overexpression of HCN2 in mice (HCN2-Tg) made hearts highly susceptible to arrhythmias induced by chronic β2-adrenergic stimulation [43]. Furthermore, HCN4 was found overexpressed in cats with hypertrophic cardiomyopathy [44], and both mRNA and protein levels of HCN4 were significantly augmented in failing human ventricles [45].

Additionally, HCN channels participate in early cardiac morphogenesis. Overexpression and inhibition of HCN4 during embryogenesis result in improper expression of key patterning genes and severely malformed hearts [46]. One common adaptation in the pathological heart is fetal reprogramming, where the adult heart expresses several genes and miRNAs that are active in the fetal stage [47], and it is possible to consider HCN4 overexpression as an indicator of fetal reprogramming in chagasic hearts, which would facilitate the genesis of arrhythmias, particularly of supraventricular ones.

It could be argued that since our experiments were undertaken during the acute phase in mice, the results cannot be extended to humans. However, the auricular arrhythmias observed in mice are commonly observed in patients [48], suggesting that our conclusions about ivabradine treatment in patients should be taken into account.

In conclusion, the present work showed, to the best of our knowledge for the first time, pharmacological and immunohistochemical evidence for HCN4 overexpression in auricles and ventricles in an EA mouse model of Chagas disease. Moreover, ivabradine action may be enhanced by this overexpression, showing a strongly marked reduction of heart rate and recovery of ST parameters during continuous treatment. Finally, the arrhythmogenic potential of ivabradine associated with the QT increase was presumably moderated by the so-called repolarization reserve, especially in the single-dose regimen, but supraventricular arrhythmias were enhanced by treatment, suggesting the need for caution when considering ivabradine for treatment of chagasic patients.

## References

[1] World Health Organization (2015). Chagas disease in Latin America: An epidemiological update based on 2010 estimates. Wkly. Epidemiol. Rec..

[2] Higuchi M.D.L., Benvenuti L.A., Martins Reis M., Metzger M. (2003). Pathophysiology of the heart in Chagas’ disease: Current status and new developments. Cardiovasc. Res..

[3] Mendoza I., Camardo J., Moleiro F., Castellanos A., Medina V., Gomez J., Acquatella H., Casal H., Tortoledo F., Puigbo J. (1986). Sustained ventricular tachycardia in chronic Chagasic myocarditis: Electrophysiologic and pharmacologic characteristics. Am. J. Cardiol..

[4] Baroldi G., Oliveira S.J., Silver M.D. (1997). Sudden and unexpected death in clinically “silent” Chagas disease. A hypothesis. Int. J. Cardiol..

[5] Fineschi V., Silver M.D., Karch S.B., Parolini M., Turillazzi E., Pomara C., Baroldi G. (2005). Myocardial disarray: An architectural disorganization related to adrenergic stress?. Int. J. Cardiol..

[6] Rodríguez-Angulo H.O., Toro-Mendoza J., Marques J.A., Concepción J.L., Bonfante-Cabarcas R., Higuerey Y., Thomas L.E., Balzano-Nogueira L., Lopez J.R., Mijares A. (2015). Evidence of Reversible Bradycardia and Arrhythmias Caused by Immunogenic Proteins Secreted by T. cruzi in Isolated Rat Hearts. PLoS Negl. Trop. Dis..

[7] Bayer-Santos E., Aguilar-Bonavides C., Rodrigues S.P., Cordero E.M., Marques A.F., Varela-Ramirez A., Choi H., Yoshida N., da Silveira J.F., Almeida I.C. (2013). Proteomic Analysis of Trypanosoma cruzi Secretome: Characterization of Two Populations of Extracellular Vesicles and Soluble Proteins. J. Proteome Res..

[8] Daliry A., Pereira I.R., Pereira-Junior P.P., Ramos I.P., Vilar-Pereira G., Silvares R.R., Lannes-Vieira J., De Carvalho A.C.C. (2014). Levels of circulating anti-muscarinic and anti-adrenergic antibodies and their effect on cardiac arrhythmias and dysautonomia in murine models of Chagas disease. Parasitology.

[9] Vicco M.H., Pujato N., Bontempi I., Rodeles L., Marcipar I., Bottasso O. (2014). A beta1-selective adrenoceptor antagonists increase plasma levels of anti-p2beta antibodies and decrease cardiac involvement in chronic progressive Chagas heart disease. Can. J. Cardiol..

[10] Altschuller M.B., Pedrosa R.C., Pereira Bde B., Correa Filho W.B., Medeiros A.S., Costa P.C.S., de Carvalho A.C.C. (2007). Chronic Chagas disease patients with sinus node dysfunction: Is the presence of IgG antibodies with muscarinic agonist action independent of left ventricular dysfunction?. Rev. Soc. Bras. Med. Trop..

[11] Chiale P.A., Ferrari I., Mahler E., Vallazza M.A., Elizari M.V., Rosenbaum M.B., Levin M.J. (2001). Differential profile and biochemical effects of antiautonomic membrane receptor antibodies in ventricular arrhythmias and sinus node dysfunction. Circulation.

[12] Portero V., Le Scouarnec S., Es-Salah-Lamoureux Z., Burel S., Gourraud J.B., Bonnaud S., Lindenbaum P., Simonet F., Violleau J., Baron E. (2016). Dysfunction of the Voltage-Gated K+ Channel beta2 Subunit in a Familial Case of Brugada Syndrome. J. Am. Heart Assoc..

[13] Beziau D.M., Barc J., O’Hara T., Le Gloan L., Amarouch M.Y., Solnon A., Pavin D., Lecointe S., Bouillet P., Gourraud J.B. (2014). Complex Brugada syndrome inheritance in a family harbouring compound SCN5A and CACNA1C mutations. Basic Res. Cardiol..

[14] Sartiani L., Romanelli M., Mugelli A., Cerbai E. (2015). Updates on HCN Channels in the Heart: Function, Dysfunction and Pharmacology. Curr. Drug Targets.

[15] Biel S., Aquila M., Hertel B., Berthold A., Neumann T., DiFrancesco D., Moroni A., Thiel G., Kauferstein S. (2016). Mutation in S6 domain of HCN4 channel in patient with suspected Brugada syndrome modifies channel function. Pflügers Arch.—Eur. J. Physiol..

[16] Stallmeyer B., Kuß J., Kotthoff S., Zumhagen S., Vowinkel K., Rinné S., Matschke L.A., Friedrich C., Schulze-Bahr E., Rust S. (2017). A Mutation in the G-Protein Gene GNB2 Causes Familial Sinus Node and Atrioventricular Conduction Dysfunction. Circ. Res..

[17] Delgado-Ramirez M., Pottosin I.I., Melnikov V., Dobrovinskaya O.R. (2010). Infection by Trypanosoma cruzi enhances anion conductance in rat neonatal ventricular cardiomyocytes. J. Membr. Biol..

[18] Balbi T., Ghimenton C., Pasquinelli G., Foroni L., Grillini M., Pierini G. (2011). Advancement in the examination of the human cardiac sinus node: An unexpected architecture and a novel cell type could interest the forensic science. Am. J. Forensic Med. Pathol..

[19] Bucchi A., Baruscotti M., DiFrancesco D. (2002). Current-dependent block of rabbit sino-atrial node I(f) channels by ivabradine. J. Gen. Physiol..

[20] Jiménez M.A.V., Nascimento J.H., Monnerat G., Maciel L., Paiva C.N., Pedrosa R.C., de Carvalho A.C.C., Medei E. (2017). Autoantibodies with beta-adrenergic activity from chronic chagasic patients induce cardiac arrhythmias and early afterdepolarization in a drug-induced LQT2 rabbit hearts. Int. J. Cardiol..

[21] Rodriguez-Angulo H., Marques J., Mendoza I., Villegas M., Mijares A., Girones N., Fresno M. (2017). Differential cytokine profiling in Chagasic patients according to their arrhythmogenic-status. BMC Infect. Dis..

[22] De Carvalho A.C., Masuda M.O., Tanowitz H.B., Wittner M., Goldenberg R.C., Spray D.C. (1994). Conduction defects and arrhythmias in Chagas’ disease: Possible role of gap junctions and humoral mechanisms. J. Cardiovasc. Electrophysiol..

[23] Lazzerini P.E., Capecchi P.L., Laghi-Pasini F., Boutjdir M. (2017). Autoimmune channelopathies as a novel mechanism in cardiac arrhythmias. Nat. Rev. Cardiol..

[24] Choy L., Yeo J.M., Tse V., Chan S.P., Tse G. (2016). Cardiac disease and arrhythmogenesis: Mechanistic insights from mouse models. Int. J. Cardiol. Heart Vasc..

[25] Waghabi M.C., Coutinho-Silva R., Feige J.J., Higuchi Mde L., Becker D., Burnstock G., de Araújo-Jorge T.C. (2009). Gap junction reduction in cardiomyocytes following transforming growth factor-beta treatment and Trypanosoma cruzi infection. Mem. Inst. Oswaldo Cruz.

[26] Cruz J.S., Machado F.S., Ropert C., Roman-Campos D. (2017). Molecular mechanisms of cardiac electromechanical remodeling during Chagas disease: Role of TNF and TGF-beta. Trends Cardiovasc. Med..

[27] Rodriguez H.O., Guerrero N.A., Fortes A., Santi-Rocca J., Girones N., Fresno M. (2014). Trypanosoma cruzi strains cause different myocarditis patterns in infected mice. Acta Tropica..

[28] Poveda C., Herreros-Cabello A., Callejas-Hernández F., Osuna-Pérez J., Maza M.C., Chillón-Marinas C., Calderón J., Stamatakis K., Fresno M., Gironès N. (2020). Interaction of Signaling Lymphocytic Activation Molecule Family 1 (SLAMF1) Receptor with Trypanosoma Cruzi Is Strain-Dependent and Affects NADPH Oxidase Expression and Activity. PLoS Negl. Trop. Dis..

[29] Elizari M.V., Chiale P.A. (1993). Cardiac Arrhythmias in Chagas’ Heart Disease. J. Cardiovasc. Electrophysiol..

[30] John R.M., Kumar S. (2016). Sinus Node and Atrial Arrhythmias. Circulation.

[31] Wu C., Dong X., Liu Q., Zhu J., Zhang T., Wang Q., Hu X., Yang Z., Li L. (2017). EP3 activation facilitates bladder excitability via HCN channels on ICCs. Biochem. Biophys. Res. Commun..

[32] Acosta C., McMullan S., Djouhri L., Gao L., Watkins R., Berry C., Dempsey K., Lawson S.N. (2012). HCN1 and HCN2 in Rat DRG neurons: Levels in nociceptors and non-nociceptors, NT3-dependence and influence of CFA-induced skin inflammation on HCN2 and NT3 expression. PLoS ONE.

[33] Hancox J.C., Melgari D., Dempsey C., Brack K.E., Mitcheson J., Ng G.A. (2015). hERG potassium channel inhibition by ivabradine may contribute to QT prolongation and risk of torsades de pointes. Ther. Adv. Drug Saf..

[34] Camm A.J., Lau C.P. (2003). Electrophysiological effects of a single intravenous administration of ivabradine (S 16257) in adult patients with normal baseline electrophysiology. Drugs R D.

[35] Frommeyer G., Weller J., Ellermann C., Bogeholz N., Leitz P., Dechering D.G., Kochhäuser S., Wasmer K., Eckardt L. (2017). Ivabradine Reduces Digitalis-induced Ventricular Arrhythmias. Basic Clin. Pharmacol. Toxicol..

[36] Melgari D., Brack K.E., Zhang C., Zhang Y., El Harchi A., Mitcheson J., Dempsey C.E., Ng G.A., Hancox J.C. (2015). hERG Potassium Channel Blockade by the HCN Channel Inhibitor Bradycardic Agent Ivabradine. J. Am. Heart Assoc..

[37] Varró A., Baczkó I. (2011). Cardiac ventricular repolarization reserve: A principle for understanding drug-related proarrhythmic risk. Br. J. Pharmacol..

[38] Sanguinetti M.C., Tristani-Firouzi M. (2006). hERG potassium channels and cardiac arrhythmia. Nat. Cell Biol..

[39] Mengesha H.G., Weldearegawi B., Petrucka P., Bekele T., Otieno M.G., Hailu A. (2017). Effect of ivabradine on cardiovascular outcomes in patients with stable angina: Meta-analysis of randomized clinical trials. BMC Cardiovasc. Disord..

[40] Niccoli G., Borovac J.A., Vetrugno V., Camici P.G., Crea F. (2017). Ivabradine in acute coronary syndromes: Protection beyond heart rate lowering. Int. J. Cardiol..

[41] Marin-Neto J.A., Simoes M.V., Ayres-Neto E.M., Attab-Santos J.L., Gallo L., Amorim D.S., Maciel B.C. (1995). Studies of the coronary circulation in Chagas’ heart disease. Sao Paulo Med. J. Rev. Paul. Med..

[42] Hofmann F., Fabritz L., Stieber J., Schmitt J., Kirchhof P., Ludwig A., Herrmann S. (2012). Ventricular HCN channels decrease the repolarization reserve in the hypertrophic heart. Cardiovasc. Res..

[43] Kuwabara Y., Kuwahara K., Takano M., Kinoshita H., Arai Y., Yasuno S., Nakagawa Y., Igata S., Usami S., Minami T. (2013). Increased Expression of HCN Channels in the Ventricular Myocardium Contributes to Enhanced Arrhythmicity in Mouse Failing Hearts. J. Am. Heart Assoc..

[44] Riesen S.C., Schober K.E., Bonagura J.D., Carnes C.A. (2013). Myocardial expression of hyperpolarization-activated, cyclic nucleotide-gated proteins in healthy cats and cats with hypertrophic cardiomyopathy. Schweiz. Arch. Tierheilkd..

[45] Stillitano F., Lonardo G., Zicha S., Varro A., Cerbai E., Mugelli A., Nattel S. (2008). Molecular basis of funny current (If) in normal and failing human heart. J. Mol. Cell. Cardiol..

[46] Udoko A.N., Johnson C.A., Dykan A., Rachakonda G., Villalta F., Mandape S.N., Lima M.F., Pratap S., Nde P.N. (2016). Early Regulation of Profibrotic Genes in Primary Human Cardiac Myocytes by Trypanosoma cruzi. PLoS Negl. Trop. Dis..

[47] Pitcairn E., Harris H., Epiney J., Pai V.P., Lemire J.M., Ye B., Shi N.Q., Levin M., McLaughlin K.A. (2017). Coordinating heart morphogenesis: A novel role for hyperpolarization-activated cyclic nucleotide-gated (HCN) channels during cardiogenesis in *Xenopus laevis*. Commun. Integr. Biol..

[48] Benchimol-Barbosa P., Barbosa-Filho J. (2009). Mechanical cardiac remodeling and new-onset atrial fibrillation in long-term follow-up of subjects with chronic Chagas’ disease. Braz. J. Med. Biol. Res..

